# Determination of Artemisinin in Bulk and Pharmaceutical Dosage Forms using HPTLC

**DOI:** 10.4103/0250-474X.51948

**Published:** 2009

**Authors:** S. P. Agarwal, A. Ali, Yashomati Dua, Shipra Ahuja

**Affiliations:** Department of Pharmaceutics, Faculty of Pharmacy, Jamia Hamdard University, New Delhi-110 062, India

**Keywords:** Artemisinin, analysis, HPTLC, dosage form

## Abstract

A new, simple, rapid, accurate and precise HPTLC method was developed. The detector response was linear for concentrations between 100-600 ng/spot (r =0.9931). The limits of detection and quantitation were 25 ng/spot and 75 ng/spot, respectively. The recovery study was carried out by standard addition method and was found to be 99.60±0.27. Statistical analysis proved that the method was precise, accurate and reproducible, and hence was suitable for the routine analysis of artemisinin.

Artemisinin is a white, odorless, crystalline powder. It is a potent antimalarial drug isolated from the plant Artemisia annua[1]. Chemically it is a sesquiterpene lactone with an endoperoxide bridge (-C-O-O-C-), which is responsible for its antimalarial activity is attributed[2]. Presently TLC[3],GC[4], HPLC with UV[5]/chemiluminescent[6]/electrochemical[7] detectors, RIA[8] and ELISA[9] methods are documented/employed for the determination of artemisinin. Therefore, An attempt was made to develop an HPTLC method, which was specific, accurate, precise and reproducible. A gift sample of artemisinin was obtained from Saokin Co-operation Ltd., Vietnam. Toluene (sulphur free), vanillin and sulphuric acid were of AR grade. All other chemicals were of HPLC grade. The instrument used in the present study was HPTLC system comprising LINOMAT V automatic sample applicator, TLC SCANNER III with WINCATS software; twin through chamber (all from CAMAG, Switzerland) and UV-1601, UV/Vis spectrophotometer, Shimadzu (Kyoto, Japan).

Following chromatographic conditions were used: stationary phase, silica gel F_254_ HPTLC precoated plates, 200 μm layer thickness, mobile phase: toluene:ethyl acetate (10:1); chamber saturation time: 30 min; Sample application: 5 mm band; separation technique: ascending; temperature: 20±5°; migration distance: 75 mm; scanning mode: absorbance; Detection wavelength: 520 nm; and source of radiation utilized: combination of D_2_ and tungsten lamps.

A stock solution of artemisinin (100 ng/μl) was prepared in methanol. Different amounts of stock solution were spotted in duplicate on TLC plate with the help of an automatic sample applicator, to obtain concentrations of 100, 200, 300, 400, 500 and 600 ng/spot of artemisinin. The plates were developed in a presaturated twin trough chamber and densitometrically scanned at 520 nm using optomechanical scanning technique. The data of peak area versus drug concentration were treated by linear least-square regression analysis.

Repeatability of sample application and measurement of peak area were assessed or established by using six replicates of the same spot (500 ng/spot) and also by scanning six spots of the same concentration (500 ng/spot). The intra and inter day precision were determined at three different concentration levels of 200, 600 and 3000 ng /spot.

Additionally, studies were carried out to determine the effect of altering various experimental conditions on the results. The variables investigated were also mobile phase composition, amount of mobile phase, plate treatment, time from spotting to chromatography and time from chromatography to scanning. The studies were also carried out at concentration levels of 200, 600 and 3000 ng/spot in triplicate.

In order to estimate the LOD and LOQ, blank methanol was spotted six times on HPTLC plate and then developed, sprayed and scanned in a similar way as that for calibration curve and then signal-to-noise ratio was determined.

LOD was determined using the equation LOD= 3.3σ/S, while LOQ was determined by employing the relationship LOQ= 10σ/S, where σ= the standard deviation of the response and S= the slope of the calibration curve.

A stock solution of marketed preparations having concentration of 100μg/ml of artemisinin was prepared. This solution was first analyzed by the proposed method. In the analyzed sample, an extra 80%, 100% and 120% of artemisinin was spiked and then the mixture was reanalyzed. The experiment was conducted in triplicate. It was done to find out the recovery of drug at different levels in the formulation.

To determine the content of artemisinin in capsules (Mekophar, Vietnam; labeled claim: 250 mg/capsule), twenty capsules were weighed and the content was finely powdered. The powder equivalent to 250 mg of artemisinin was weighed accurately and extracted with 40 ml of ethanol. The solution was sonicated for 30 min and volume was made to 100 ml with ethanol. The resulting solution was centrifuged at 3000 rpm for 5 min and filtered twice. Final concentration of 2500 ng/μl was obtained and μl of the solution was spotted on the plate and developed, sprayed and scanned optomechanically. The experiment was repeated six times.

Pure solvents of different selectivities, like methanol, diethyl ether, toluene, chloroform, acetonitrile etc. and their mixtures in different proportions were tried as mobile phase for the development of chromatogram. The mobile phase, which was finally found suitable, was toluene:ethyl acetate (10:1). Since artemisinin is a non UV-absorbing compound, it was converted into a violet colored UV-absorbing compound by using vanillin (1% w/v) and sulphuric acid (5% v/v) in ethanol as derivatizing agent. Densitometric quantitation was carried out in the absorbance mode at 520 nm and symmetrical, well resolved and well defined peaks were obtained. The standard plot of artemisinin (fig. 1) showed good linear relationship with respect to peak area, with coefficient of correlation values of r=0.9967 over the concentration range 100-600 ng per spot.

**Fig. 1:**
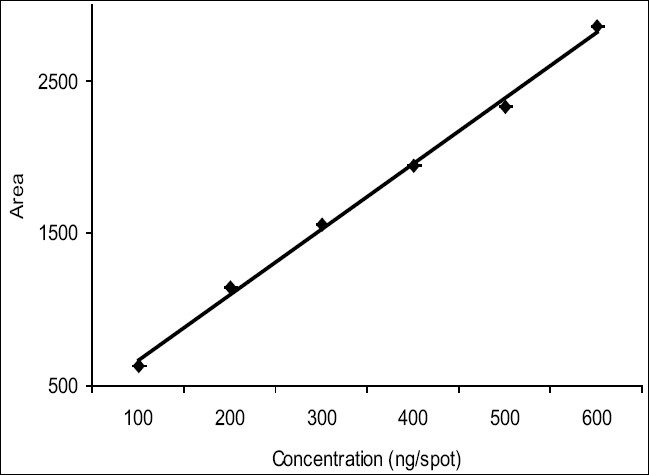
Standard plot of artemisinin (y = 431.7× + 233.72., R^2^=0.9967)

The % RSD values 0.33 and 0.36 were observed for intra and interday precision studies respectively (Table 1). The low values of % RSD obtained after introducing small changes in mobile phase composition and volume (Table 2) were indicative of the robustness of the method.

**TABLE 1:** INTRA-AND INTER-DAY PRECISION OF HPTLC METHOD FOR ARTEMISININ

Amount (ng/spot)	Intra-day precision			Inter-day precision		
		
	Mean area	SD	% RSD	Mean area	SD	% RSD
200	859	1.90	0.22	918.96	2.69	0.30
600	1658.54	3.80	0.21	1718.25	2.91	0.17
3000	7320.65	41.31	0.56	7529.83	32.76	0.61

% RSD is displayed for intra and interday precision studies. (n=6)

**TABLE 2:** ROBUSTNESS OF THE METHOD FOR ARTEMISININ

Parameters	% RSD			Mean % RSD
		
	200 ng/μl	600 ng/μl	3000 ng/μl
Mobile phase composition	0.41	0.28	0.65	0.45
Amount of mobile phase	0.30	0.20	0.58	0.36
Plate treatment	0.35	0.25	0.50	0.37
Time from spotting to chromatography	0.50	0.36	0.68	0.51
Time from chromatography to scanning	0.45	0.33	0.52	0.42

% RSD was observed after introducing small changes in mobile phase composition and volume etc. (n=3)

The limit of detection and limit of quantitation for artemisinin were calculated to be 25 ng/spot and 75 ng/spot respectively. The accuracy of the method was evaluated by % recovery (standard addition method) of the drug. The average recovery was found to be 99.60 with average % RSD. value of 0.27 (Table 3). The developed HPTLC method was applied to the analysis of artemisinin in capsule dosage form (Table 4). The mean % recovery value of 99.34 was obtained with % RSD value of 0.19. The values of S.D. or % RSD and coefficient of correlation were within the prescribed limit of 2% showing the high precision of the method. Hence, this indicates the suitability of the method for the routine analysis of artemisinin in bulk and pharmaceutical dosage forms. The developed HPTLC method was compared with HPLC method. The HPLC[10] method gave a mean % recovery of 99.68 ± 0.18, which agrees with values obtained by the proposed method.

**TABLE 3:** RECOVERY STUDIES FOR ARTEMISININ

Excess drug added to analyte (%)	Theoretical content	% recovery	% RSD
80	90	98.75	0.33
100	100	99.60	0.21
120	110	100.45	0.28

The accuracy of the method was evaluated by % recovery (standard addition method) of the drug. (n=3)

**TABLE 4:** ANALYSIS OF MARKETED FORMULATION OF ARTEMISININ BY HPTLC AND HPLC METHOD

Throretical content (mg/capsule)	Amt. of drug recovered		% recovery	
		
	HPTLC	HPLC	HPTLC	HPLC
250	248.93	249.44	99.57	99.77
250	248.30	249.58	99.32	99.83
250	247.80	248.56	99.12	99.42

The developed HPTLC method was compared with HPLC method. The mean % recovery after analysis by HPLC agrees with values obtained by developed HPTLC method. (n=3)
